# Shear Wave Elastography: A New Noninvasive Tool to Assess the Intensity of Fibrosis of Irradiated Salivary Glands in Head and Neck Cancer Patients

**DOI:** 10.1155/2014/157809

**Published:** 2014-08-17

**Authors:** Jarosław Kałużny, Tomasz Kopeć, Ewelina Szczepanek-Parulska, Adam Stangierski, Edyta Gurgul, Marek Ruchała, Piotr Milecki, Małgorzata Wierzbicka

**Affiliations:** ^1^Department of Otolaryngology, Head and Neck Surgery, Poznan University of Medical Sciences, 49 Przybyszewskiego Street, 60-355 Poznan, Poland; ^2^Department of Endocrinology, Metabolism and Internal Medicine, Poznan University of Medical Sciences, 49 Przybyszewskiego Street, 60-355 Poznan, Poland; ^3^Great Poland Oncology Center, Department of Electroradiology, Poznan University of Medical Sciences, 15 Garbary Street, 61-866 Poznan, Poland

## Abstract

The aim of the study was to assess salivary gland parenchyma by means of sonoelastography in patients irradiated for head and neck squamous cell carcinoma (HNSCC). The studied group consisted of 52 patients after radiotherapy (RT) and 54 healthy volunteers. All of the former were treated for advanced larynx (40), oropharynx (9), or maxilla (3) squamous cancers and suffered from chronic dryness. Ultrasonography (US) and elastography (ES) were performed, as well as an assessment of the amount of saliva and Common Terminology Criteria for Adverse Events (CTCAE) scale. There was a statistical difference between ES values in the RT group and in the controls for parotid glands (41.7 kPa versus 26.03 kPa, *P* = 0.0018) and for submandibular glands (37.6 kPa versus 22.4 kPa; *P* = 0.005). There was a significant correlation between the CTCAE scores and objective saliva amount (*P* = 0.0005), and the median amount of saliva in the examined group was lower than in the reference group (1.86 g versus 2.75 g, *P* = 0.0006). In conclusion sonoelastography adds a new parameter to ultrasonography in “one touch examination” and may be a useful tool for major salivary gland evaluation during the radiotherapy course and follow-up period.

## 1. Introduction

Radiotherapy (RT) is the mainstay in head and neck squamous cell cancer (HNSCC) treatment. Radiation-induced salivary gland injuries are very common and occur in up to 80% of cases [1, 2]. Two aspects of this problem can be listed: profound salivary gland hypofunction (diminished salivary flow) and xerostomia (subjective sensation of a dry mouth). Since the National Institutes of Health Consensus Development Conference on the Oral Complications of Cancer Therapies (1989) salivary gland hypofunction and xerostomia have been considered to be basic sequelae of radiotherapy. What is important is that reduced gland function and parallel oral health-related quality of life decrease and are typical for both acute and chronic phases of sialoadenitis [3, 4]. One of the most important tools in xerostomia assessment is the Common Terminology Criteria for Adverse Events (CTCAE v. 4.0) scale, which indicates the intensity of a patient's subjective complaints.

The apparent radiosensitivity of the salivary glands is still a “radiobiology enigma,” because their cells are highly differentiated, have a slow turnover, and should therefore not be particularly radiosensitive [5]. The histological changes in the gland tissue during irradiation are not accessible* in vivo* [6]. The ongoing trials conducted to develop radioprotective agents for salivary glands will probably improve gland parenchyma assessment [7]. The diagnostic techniques for salivary gland evaluation are well established; however efforts to introduce new methods are still being made.

Elastography is a term referring to the imaging technique that assesses tissue elasticity* in vivo*. Qualitative real time ultrasound elastography (ES) is a newly available modality, overlapping elasticity to the grey-scale images. Sonoelastography was first used for predicting malignancy in thyroid, prostate, pancreatic, and breast nodules [8–10]. The usefulness of this novel technique was demonstrated in the evaluation of the status of thyroid parenchyma in de Quervain thyroiditis and liver fibrosis in chronic hepatitis and the assessment of obstructive diseases of the salivary glands [11–14]. The results of elastographic evaluation of parotid and submandibular focal gland masses were then published [15–18].

The question arises of whether elastography can deliver additional information about the irradiated salivary gland and whether this method could therefore help in better qualification of patients for subsequent intensive postradiation treatment. The subject of our research implements the NIH Development Consensus Conference recommendation to develop future research applicable to the management strategies of salivary gland hypofunction and xerostomia [3]. The authors hypothesize that sonoelastography introduced to salivary gland routine repeatable imaging may constitute an objective measure that would correlate with dryness of the mouth. The aim of the study was to assess the features of salivary gland parenchyma by means of high resolution sonoelastography in patients irradiated for HNSCC and to correlate the findings with objective saliva amount and subjective complaints caused by irradiation-induced sialadenitis.

## 2. Material and Methods

Informed patient consent and Bioethical Committee of Poznan University of Medical Sciences approval (nr 494/12) were obtained. To address the research purpose the investigators designed and implemented the prospective cross-sectioned study of patients with HNSCC aged 48–77, mean 63, median 62 years. Fifty-four healthy volunteers aged 43–78 years, mean 60, constituted the reference group for the elastography values. The patients were matched for age and gender with the control group. To be included in the study sample patients needed to have HNSCC treated with a full course of radiotherapy. All 52 patients were treated for advanced larynx (40), oropharynx (9), and maxilla (3) squamous cancers and suffered from chronic dryness. Forty-three out of the 52 patients were operated on, and adjuvant RT was administered (dose 60–64 Gy); 9/52 were treated by primary radiochemotherapy (RT/CT) (dose 62–64 Gy + 3 Cisplatin courses). Two RT modalities were applied: 33/52 received conventional external beam radiotherapy, and 19/52 received IMRT.

The intensity of xerostomia was assessed immediately after RT and during follow-up after 6, 12, and 24 months. Subjective complaints after RT concerning dry mouth were assessed twice (after RT and during follow-up) in the CTCAE scale. According to this scale, dry mouth sensation is divided into 3 categories: (1) symptomatic (e.g., dry or thick saliva) without significant dietary alteration: unstimulated saliva flow >0.2 mL/min; (2) moderate symptoms: oral intake alterations (e.g., copious water, other lubricants, diet limited to purees and/or soft, moist foods), unstimulated saliva 0.1 to 0.2 mL/min; and (3) inability to adequately aliment orally: tube feeding or total parenteral nutrition (TPN) indicated, unstimulated saliva <0.1 mL/min. Objective salivary volume measurement at follow-up visit encompassed total parotid and submandibular gland secretion. Saliva was collected after 2 minutes of chewing, and the amount was given in grams (g).

**Figure 1:**
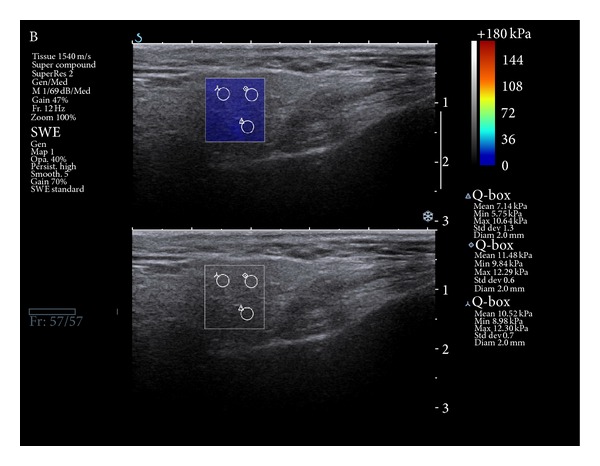
Sonoelastography picture: normal parotid gland tissue is marked as blue, which confirms parenchyma low stiffness.

Ultrasonography and sonoelastography as a real time “one touch examination” using Aixplorer (SuperSonic Imagine) were performed. Two operators with four years' experience in the technique conducted the examination. The patients were examined in the supine position. Transverse scans of salivary glands were acquired. A high resolution grey scale US 5–15 MHz linear array transducer was placed both in the middle of the unaffected gland and in heterogenic areas, in the case of alterations in the US picture. Elasticity values in kiloPascal's (kPa) were collected; the minimum, maximum, and mean values at 3 measurement points in different parts of the glands were taken (Figure 1). The US images were categorized as isoechogenic (50 patients) and with marked fibrosis (2 patients).

The following variables were analyzed: localization of HNSCC primary site, time period from RT. Objective amount of saliva and subjective complaints in the CTCAE scale were taken into consideration. The primary outcome variable for this research was the stiffness of salivary gland parenchyma, measured in kPa. Statistical analysis was performed by means of Spearman's rank correlation coefficient, Levene's, Newman-Keuls, Kruskal-Wallis, and Mann-Whitney tests.

## 3. Results

In conventional ultrasonography, in all 52 patients except two the submandibular and parotid glands were isoechogenic, without any alterations. In two patients moderate fibrosis was found.

Directly after radiotherapy 16, 29, and 2 patients gained CTCAE scores of 1, 2, and 3, respectively; 5 had no complaints (score “0”). At the follow-up visit 32 and 7 patients had CTCAE scores of 1 and 2; 13 had no dryness (score “0”). The variability of complaints dependent on the time that elapsed from RT was not statistically significant. Taking into consideration the type of RT, the difference between the CTCAE scores in 9 primary (all staged as 2) and 43 adjuvant RT (stages 0, 1, 2, and 3 in 5, 16, 20, and 2, resp.) was significant immediately after treatment (*P* = 0.02). The difference after one year of follow-up was not noticeable. There was no statistical difference between CTCAE scores in 19 cases after IMRT (1, 4, 14, and 0 patients gained scores of 0, 1, 2, and 3, resp.) and 33 subjects after conventional RT (4, 12, 15, and 3 patients, resp.). The localization of the tumor had no impact on CTCAE scale result. There was no difference between the 37 patients who had undergone RT for the lateral (lymph nodes regions) and the 15 irradiated exclusively for central neck fields (the primary tumor area).

Objective saliva amount collected from the examined group ranged from 0.4 g to 4.75 g (mean 2.15 g, median 1.85 g) and from the control group 2.75 g (*P* = 0.0006). Time from RT had no impact on the mean amount of saliva, which was equal to 1.6 g, 2.3 g, and 2.2 g (median 1.4 g, 1.7 g, 2.1 g) after 6, 12, and 24 months, respectively. There was no statistical difference between 9 primary (mean 1.6 g, median 1.6 g) and 43 adjuvant RT (mean 2.27 g, median 1.9 g) patients. Regarding particular primaries, saliva amount was statistically lower for patients irradiated for larynx (mean 2.13 g, median 1.87 g) than maxilla (mean 4.4 g, median 5.5) (*P* = 0.0012) and even lower for the oral cavity (mean 1.5 g, median 1.6 g) (*P* = 0.0003); the patients irradiated for tonsil cancer turned out to be the most prone to dryness. There was no difference between patients who had undergone RT for both neck fields (2.18 g), one side of the neck (2.02 g), central compartment (2.2 g), and exclusively for the primary focus outside the neck (2.0 g).

There was a significant correlation between the obtained subjective CTCAE scale scores and objective saliva amount directly after radiotherapy (*P* = 0.0005) and in follow-up (*P* = 0.0000). The mean amount of saliva directly after radiotherapy was 3.5 g, 2.5 g, 1.8 g, and 0.66 g (median 2.93 g, 2.07 g, 1.60 g, and 0.66 g) for the patients with scores 0, 1, 2, and 3; during the follow-up the saliva amount was 3.4 g, 2.0 g, and 0.55 g (median 2.9 g, 1.80 g, 0.53 g) for scores 0, 1, and 2.

**Figure 2:**
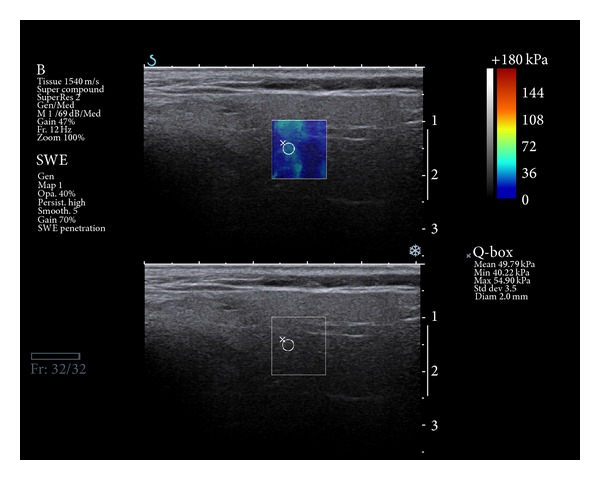
Sonoelastography picture: irradiated parotid gland tissue is marked in the green pattern, which confirms parenchyma high stiffness.

There was a statistical difference between elastography values in the irradiated group and in controls in terms of parotid glands (41.7 kPa versus 26.0 kPa; *P* = 0.0018) and submandibular glands (37.6 kPa versus 22.4 kPa; *P* = 0.005). Sonoelastography results of 52 HNSCC patients after RT are shown in Table 1. The values in kPa were taken from the 3 different areas of parenchyma. The values ranged from 4 kPa to 160 kPa. In fact they differed dramatically, although the parenchyma was sonographically isoechogenic. The mean and the median values of the changed parenchyma for the whole group (representing the average highest stiffness value) were 39.6 kPa and 34.9 kPa, respectively; these two variables were taken into consideration in further analysis. The mean and the median value for both parotid glands in the examined group were 41.7 kPa and 40 kPa (Figure 2), while for both submandibular glands they were 37.6 kPa and 34.5 kPa, respectively.

Stiffness of parotid and submandibular glands was more pronounced in patients with primaries localized in the oral cavity (mean 50.7 kPa, median 47.5 kPa) than the larynx (mean 39.73 kPa, median 34.6 kPa) or maxilla (mean 41.2 kPa, median 39.5 kPa) but did not achieve statistical significance. The impact of time since RT for elasticity values is unclear. Directly after RT, in 6, 12, and 24 months the mean elastography values were 34.2 kPa, 36.6 kPa, 46.0 kPa, and 39.7 kPa, respectively, with differences that were not significant.

The mean elasticity values did not significantly differ between the IMRT technique and conventional RT for parotid glands (44.4 kPa versus 40 kPa) and submandibular glands (34.5 kPa versus 39.4 kPa). Noticeable disparities between the primary treatment and adjuvant RT for parotid glands (46.2 kPa versus 33.6 kPa) and submandibular glands (39.5 kPa versus 30.3 kPa) were noted; nevertheless the scores did not achieve statistical significance. There was no correlation between the CTCAE score (subjective complaints) and elastography values. Patients with scores 0, 1, 2, and 3 received mean elastographic values 37.3 kPa, 45.4 kPa, 37.1 kPa, and 35.5 kPa, respectively, from all major glands. Finally, there was no correlation between the amount of saliva and stiffness.

## 4. Discussion

The clinical problem, radiation-induced sialadenitis, causing the disabled function of salivary glands and dryness syndrome, is the most common cause of the decrease in life quality after external radiotherapy in the majority of HNSCC patients [19], and thus the significance of appropriate diagnostics is crucial. Reliable radiological methods to evaluate xerostomia are also necessary; ultrasonography, computed tomography, and magnetic resonance are the methods of choice in imaging the status of parenchyma [20]. Sialography, sialoendoscopy, and MR sialography are useful in Stensen's duct visualization. The measurements of saliva amount and examination of sediment indicate the secretory function of the glands [21]. Nonetheless, binding the parenchyma features with secretory abilities is difficult. The objective methods, scintiscan, single-photon emission computed tomography (SPECT) [22], and dynamic MR sialography [23, 24], are more difficult to perform, especially in acute phase of radiation sialadenitis. One currently available technique that can visualize the major salivary glands and does not require any invasive procedures, like cannulation and contrast media infusion, is MR sialography [25]. In addition, noninvasiveness of this method is suitable for chronologic observation of gland hypofunction. However, for the prediction of radiation-induced xerostomia these techniques have disadvantages, including unavailability, high cost, and long duration of the test [1]. Katz et al. have shown that ultrasonography with high resolution transducers, routinely used in neck node monitoring in head and neck follow-up, is a highly sensitive method of evaluating the major salivary glands [26]. Nevertheless, in chronic inflammation the glandular modifications seen on US are often less prominent than in acute diseases; unfortunately in this technique no functional data are available. In the early, acute phase of radiation sialadenitis the gland is anechoic [26]. Furthermore, if later glandular atrophy occurs, marked reduction of volume with higher echogenicity appears, but still the sonographic picture does not represent the secreting ability of the glands, and thus its correlation with subjective patient complaints is not possible. According to Zenk [27], in the first, acute phase the gland is swollen and anechoic. If later glandular atrophy occurs, a marked reduction of volume with higher echogenicity may be found. Our results confirmed that in fact the sonographic picture does not reflect the secreting ability of the glands, and discrete sonographic changes rarely correspond with the subjective patient complaints. In all our patients except two, normoechogenic tissue was visualized. To conclude, the imaging possibilities of ultrasonography in radiation-induced sialadenitis are not in our opinion sufficient, and the usefulness of this method in predicting the xerostomia is very limited.

Elastography, a new development in ultrasound imaging, may deliver additional information about parenchyma status [28]. Elastography is a term referring to an imaging technique that assesses tissue elasticity* in vivo*. It provides a new insight into the evaluation of gland parenchyma that may be translated into clinical applications in the field of irradiation consequences in HNSCC treatment, but there are no literature data concerning this issue. The application of sonoelastography in our clinical practice tries to answer the need for the development of an accurate, quantifiable, and reproducible assessment of salivary glands in HNSCC patients. Tissue elasticity in salivary glands probably reflects a combination of factors, including the quantity and quality of salivary glandular tissue, and other components, such as fatty tissue or fibrosis, which may be increased in irradiated glands.

The presented study confirmed a correlation between tissue elasticity and postradiative salivary gland hypofunction. The most important finding in this study was the statistical difference in parenchymal elasticity between the RT and the reference group. The mean values from healthy volunteers (22 kPa for submandibular and 26 kPa for parotid) were statistically lower than in irradiated glands (38 kPa and 42 kPa). Unfortunately, we were unable to find correlations between the specified classes inside the RT group. Elasticity values did not correlate with CTCAE score, which can probably be explained by the small number of patients in particular the subgroups. As the time in radiotherapy plays a great role in radiobiological processes and patients' wellbeing, we pointed this out separately. The biggest difference in the mean elastography values was directly after RT (34.2 kPa) and after 12 months (46 kPa); in the further follow-up (24 months) the value became lower (40 kPa), but this trend needs further examination. The second issue is the localization of the irradiated primary. The amount of saliva indicated that patients irradiated for tonsil cancer were most liable to experience dryness. However, this fact was not reflected in CTCAE scores. Stiffness in the parotid and submandibular glands was higher in patients with primaries localized in the oral cavity (50.7 kPa and 38.55 kPa) as compared to the larynx (39.73 kPa and 34.6 kPa) and maxilla (41.2 kPa, 30 kPa), but the differences did not reach statistical significance. Similarly, the mean elastography value for the glands on the irradiated side of the neck was 40.64 kPa, and for the nonirradiated side it was 35.19 kPa (parotid 43.2 kPa versus 36.8 kPa, submandibular 38.4 kPa versus 33.5 kPa, resp.), and the trend is noticeable.

Unfortunately, our study and the method itself have some limitations. Xerostomia refers to salivary secretory quantity. It does not follow the elasticity of the glandular parenchyma. Based on these facts we can assume that the elasticity of salivary tissue depends on many antagonistic factors (e.g., fatty replacement and fibrosis) and does not correspond directly to salivary function. Increased fibrosis, which is difficult to predict in irradiated glands, may or may not influence the secretory properties of the gland. There is also a wide overlap in the values of elasticity obtained from healthy and irradiated parenchyma; therefore, it is difficult to indicate cut-off values differentiating between normal and affected glands. The number and size of regions of interests need also to be standardized. The limitation of our group is quite heterogeneous in the character of the studied patients and in the prospective assessment of all studied subjects in every point of the follow-up that were not always available. Larger prospective studies are needed to fully assess the changes of elasticity during the follow-up after radiation therapy.

To conclude, this study suggests that sonoelastography adds a new parameter to the grayscale ultrasound images in “one touch examination” and may be a useful tool for salivary gland evaluation during the radiotherapy course and the follow-up. In spite of the fact that this method has not provided detailed information about the internal architecture and morphological changes of the gland, increased elasticity corresponds to the radiobiological effect and may potentially identify individuals more liable for sialadenitis during RT course.

## Figures and Tables

**Table 1:** Elastography values in kPa—summary.

		Elastography values (kPa)							
	Number of patients	Parotid gland			Submandibular gland			Mean parotid + submandibular	*P* value
		Mean	Median	St. dev.	Mean	Median	St. dev.		
Type of RT									
Primary RT	9	46.2	23	41.1	39.5	43.5	23.3	42.8	0.58
Adjuvant RT	43	33.6	32	20.0	30.3	25	20.8	32.0	
IMRT	19	44.4	33.5	33.2	34.5	33.5	21.8	39.4	0.92
Conventional	33	40.2	41.5	23.6	39.4	30	30.1	39.8	
Tumour localization									
Larynx	40	39.7	34.5	25.0	38.5	31.0	29.4	39.1	0.81
Oropharynx	9	50.7	47.5	38.2	35.6	30.0	23.4	43.4	
Maxilla	3	41.1	39.5	16.8	30.2	25.5	18.6	35.6	
RT fields									
Lateral	37	43.3	41	26.8	36.8	34.0	21	40.2	0.73
Only for primary	15	37.7	35.5	17.3	38.8	35.5	24.9	38.2	
Time from RT									
0	5	45.8	17	51.0	22.6	16	18.0	34.2	0.56
6	7	43.5	39.5	27.6	29.8	30	15.4	36.6	
12	7	44.3	39.5	24.0	47.7	54	23.9	46.0	
24	33	40.17	41.5	23.7	39.4	30	30.1	39.7	
RT group	52	41.7	40.7	24.4	37.6	34.5	22	39.6	0.00
Control group	54	26.0	25.2	8.6	22.4	21	7.5	24.2	
